# Thyroid Involvement in Laryngeal Squamous Cell Carcinoma—Should it be Spared?

**DOI:** 10.1002/oto2.70312

**Published:** 2026-10-13

**Authors:** Francisco J. Garratón‐Gutiérrez, Bianca Bonafont‐Reyes, Javier A. Vilá Ortiz, Antonio Burés‐Rodríguez, Mario Corona‐Ruíz, Jeamarie Pascual‐Marrero

**Affiliations:** ^1^ Department of Otolaryngology Head & Neck Surgery University of Puerto Rico, School of Medicine San Juan Puerto Rico USA; ^2^ Department of Surgery University of Puerto Rico, School of Medicine San Juan Puerto Rico USA; ^3^ Department of Otolaryngology—Head & Neck Surgery Massachusetts Eye and Ear Infirmary, Harvard Medical School Boston Massachusetts USA; ^4^ Department of Otolaryngology Veteran's Affairs Medical Center San Juan Puerto Rico USA

**Keywords:** Hispanic, hypocalcemia, laryngeal squamous cell carcinoma, thyroidectomy, thyroid gland invasion, total laryngectomy

## Abstract

**Objectives:**

To determine the prevalence and predictors of thyroid gland invasion in Hispanic patients with laryngeal squamous cell carcinoma undergoing total laryngectomy and to assess thyroidectomy‐associated comorbidities.

**Study Design:**

Retrospective cross‐sectional review.

**Setting:**

University District Hospital, Puerto Rico Medical Center, San Juan, PR.

**Methods:**

Records of adults with laryngeal squamous cell carcinoma who underwent total laryngectomy from 2009 to 2022 were reviewed. Tumor characteristics were correlated with histopathologic thyroid gland invasion, and postoperative hypocalcemia was documented. Categorical variables were compared using Fisher's exact or Pearson's chi‐square tests, with effect size reported by Cramér's *V*; continuous variables were analyzed with the Wilcoxon‐Mann‐Whitney test.

**Results:**

Of 105 patients, 8 (7.6%) had thyroid gland invasion. Tumors with invasion were larger (5.0 ± 1.3 vs 3.3 ± 1.4 cm; *P* = .003) and were associated with thyroid cartilage invasion (100% vs 54.6%; *P* = .020). Patients with invasion underwent total thyroidectomy more often (75.0% vs 28.9%; *P* = .013). Postoperative hypocalcemia occurred in 41.9% (44/105) of patients overall and was more frequent after total than after partial thyroidectomy (79.4% vs 23.9%; *P* < .001). Intravenous calcium replacement was required in 19.0% (20/105) of patients, more often after total thyroidectomy (47.1% vs 5.6%; *P* < .001).

**Conclusion:**

Thyroid gland invasion was uncommon but associated with larger tumors and thyroid cartilage invasion. Total thyroidectomy carried a higher risk and greater severity of hypocalcemia. These findings support selective thyroid management during total laryngectomy and provide evidence from an underrepresented Hispanic population.

Over the last several decades, recommendations regarding thyroid gland excision during total laryngectomy for laryngeal squamous cell carcinoma (LSCC) have evolved considerably. In 1955, Ogura advocated for ipsilateral hemithyroidectomy among all patients undergoing total laryngectomy,^1^ and in 1973, Harrison expanded these recommendations by including frozen section analysis of the contralateral thyroid lobe in anterior commissure tumors and total thyroidectomy for postcricoid or subglottic disease.^2^ More recently, the 2016 National Comprehensive Cancer Network (NCCN) guidelines listed thyroidectomy with neck dissection as part of the routine management of advanced LSCC.^3^

However, contemporary literature demonstrates that thyroid gland invasion (TGI) is relatively uncommon, with reported rates between 1% and 15%.^4^ Furthermore, the morbidity associated with thyroidectomy—including hypothyroidism and hypocalcemia—is substantial. Gal et al reported hypothyroidism in 23% of patients after laryngectomy,^5^ while Negm et al found thyroid or parathyroid dysfunction in more than one‐third of cases.^6^ Basheeth et al observed hypocalcemia in 32% of patients, with 7% developing permanent hypocalcemia.^7^

These complications extend beyond laboratory abnormalities: hypocalcemia can cause tetany, seizures, and life‐threatening arrhythmias, while hypothyroidism contributes to impaired wound healing, cardiovascular dysfunction, and, in rare cases, myxedema coma. The frequency and severity of these endocrine complications underscore the importance of identifying patients who truly benefit from thyroidectomy during total laryngectomy.^7^

To address this, many investigators have studied potential predictors of thyroid invasion. Proposed factors include primary tumor site, subglottic extension, thyroid cartilage involvement, radiologic evidence of extralaryngeal spread, and intraoperative findings of anterior framework invasion. Early anatomic studies by Lam^8^ and Yeager and Archer^9^ described the pathways of anterior and inferior tumor spread through the cricothyroid membrane and weak points in the thyroid cartilage, helping explain why subglottic and transglottic tumors are more likely to invade the thyroid gland.

Despite consistent evidence of low invasion rates, identifiable predictive factors, and significant postoperative morbidity, standardized guidelines and protocols for thyroid management in total laryngectomy remain lacking. Earlier series were often limited by small sample sizes, but larger contemporary studies have shown greater consistency in risk factors, strengthening the rationale for risk‐adapted thyroid management. In 2022, El‐Sebai et al studied the impact of thyroid gland preservation on locoregional tumor control in a prospective cohort study and found no statistically significant difference between thyroid preservation and thyroid‐sacrificing groups regarding tumor recurrence over a 5‐year follow‐up period.^10^ There is now a broader consensus that TGI is not an inherent feature of LSCC and that routine thyroidectomy for all laryngectomies is likely unnecessary.

The objective of this study was to evaluate the incidence of TGI in a Hispanic population with LSCC undergoing total laryngectomy, to stratify tumor characteristics associated with thyroid involvement, and to assess postoperative complications associated with thyroidectomy. We hypothesized that clinicopathologic features could help identify patients at higher risk of thyroid invasion and thereby guide selective thyroidectomy, reducing the burden of long‐term endocrine complications.

## Methods

### Patients and Data Collection

We conducted a retrospective cross‐sectional review of 105 patients with LSCC who underwent total laryngectomy with concomitant hemithyroidectomy or total thyroidectomy at the University District Hospital, Puerto Rico Medical Center, between 2009 and 2022. Patients who underwent laryngectomy for non‐squamous cell pathology were excluded.

Demographic variables (age, sex), tumor characteristics (histologic grade, tumor size, primary site, margins, TNM stage), and pathologic features (perineural invasion, lymphovascular invasion, nodal status, thyroid cartilage involvement, and TGI) were extracted from medical records. Surgical variables included extent of thyroidectomy (partial vs total). Postoperative outcomes included hypocalcemia requiring inpatient management, categorized as oral versus intravenous calcium supplementation. All data were de‐identified, and the study protocol was approved by the University of Puerto Rico Institutional Review Board (Protocol Number: 2402195417R001), which waived informed consent due to the retrospective design.

### Variable Definitions

To ensure that all reported frequencies derive from a single, reproducible coding of the source record, the following definitions were applied uniformly. The final analytic cohort comprised 105 patients, and every percentage reported in this manuscript, in the tables, and in the figures is calculated on this cohort unless a variable‐specific denominator is stated. Pathologic T category was abstracted from the final pathology stage; 10 specimens were reported as T4 without an a/b subcategory and are presented separately in Table 1 but grouped with T4 for analysis. Laryngeal subsite involvement was recorded separately for the right and left supraglottis, glottis, and subglottis; a subsite was considered involved if either side was involved, and disease was classified as bilateral if any subsite was involved on both sides. Transglottic extension was defined as tumor involving both the supraglottis and the glottis (ie, crossing the laryngeal ventricle). Because vertical spread toward the inferior laryngeal framework has been implicated in extralaryngeal extension,^8^, ^9^ involvement of all 3 laryngeal levels (supraglottis, glottis, and subglottis) was analyzed as a separate, prespecified pattern. Partial thyroidectomy comprised hemithyroidectomy and partial hemithyroidectomy; total thyroidectomy comprised removal of both lobes. Histologic grade was abstracted verbatim from the pathology report and coded into 4 ordered categories (well, well to moderately, moderately, and moderately to poorly differentiated); reports that recorded only descriptive terms without a grade were classified as grade not specified. Perineural and lymphovascular invasion were not reported in every pathology report, and denominators for these variables reflect documented cases only. Postoperative hypocalcemia was defined as hypocalcemia requiring inpatient calcium supplementation, and severity was categorized by whether intravenous replacement was required.

**Table 1:** Clinicopathologic Characteristics of 105 Patients With Laryngeal Squamous Cell Carcinoma Who Underwent Total Laryngectomy

Characteristic	Value (N = 105)
Age, y	
Median (range)	62 (44‐87)
Mean	63.4
Sex	
Male	102 (97.1)
Female	3 (2.9)
Histologic type	
Conventional squamous cell carcinoma	103 (98.1)
Sarcomatoid squamous cell carcinoma	2 (1.9)
Histologic grade (conventional SCC, n = 103)	
Well differentiated	46 (44.7)
Well to moderately differentiated	24 (23.3)
Moderately differentiated	25 (24.3)
Moderately to poorly differentiated	1 (1.0)
Grade not specified in report	7 (6.8)
Primary tumor size, cm	
Mean ± SD	3.5 ± 1.5
Median (range)	3.0 (1.0‐8.0)
Pathologic T category	
T1	8 (7.6)
T2	20 (19.0)
T3	21 (20.0)
T4a	46 (43.8)
T4, subcategory not specified	10 (9.5)
T1‐T2 (combined)	28 (26.7)
T3‐T4 (combined)	77 (73.3)
Nodal status	
Negative	41 (39.0)
Positive	23 (21.9)
Undocumented	41 (39.0)
Distant metastasis	
Present	1 (1.0)
Laryngeal subsite involvement	
Supraglottis	78 (74.3)
Glottis	97 (92.4)
Subglottis	18 (17.1)
Laterality of laryngeal involvement	
Bilateral	80 (76.2)
Unilateral	25 (23.8)
Vertical extension	
Transglottic (supraglottis + glottis)	70 (66.7)
All three laryngeal levels	12 (11.4)
Margin status	
Positive	22 (21.0)
Negative	83 (79.0)
Perineural invasion (documented, n = 94)	
Present	31 (33.0)
Absent	63 (67.0)
Lymphovascular invasion (documented, n = 101)	
Present	26 (25.7)
Absent	75 (74.3)
Thyroid cartilage invasion	
Present	61 (58.1)
Absent	44 (41.9)
Thyroid gland invasion	
Present	8 (7.6)
Absent	97 (92.4)
Extent of thyroidectomy	
Partial (hemithyroidectomy)	71 (67.6)
Total	34 (32.4)
Postoperative hypocalcemia	
Present	44 (41.9)
Managed with oral supplementation only	24 (22.9)
Required intravenous calcium replacement	20 (19.0)

Values are n (%) unless otherwise indicated. Percentages are calculated on N = 105 unless a variable‐specific denominator is stated. Perineural and lymphovascular invasion were not reported in every pathology report; denominators reflect documented cases only. T category was abstracted from the final pathologic stage; 10 specimens were reported as T4 without an a/b subcategory. Transglottic extension was defined as tumor involving both the supraglottis and the glottis. Partial thyroidectomy includes hemithyroidectomy and partial hemithyroidectomy.

Abbreviations: SCC, squamous cell carcinoma; SD, standard deviation.

### Statistical Analysis

Categorical variables were summarized as frequencies and percentages, and continuous variables as means with standard deviations, medians, and ranges. Associations between TGI and clinicopathologic features were assessed with Fisher's exact test, with Cramér's *V* used to describe the strength of association. Continuous variables were analyzed with the Wilcoxon‐Mann‐Whitney rank‐sum test, with the probability of superiority reported when significant. Pearson's chi‐square test was applied to categorical comparisons with sufficient expected counts, including extent of thyroidectomy and hypocalcemia. Because only 8 invasion events were observed, all analyses of predictors of TGI are exploratory and hypothesis‐generating, and multivariable adjustment was not attempted. Odds ratios are not reported for predictors of TGI because zero and near‐zero cell counts render them unstable; Cramér's *V* is reported instead. All tests were two‐sided, with *P* ≤ .05 considered statistically significant. Analyses were conducted using Stata version 14.2 (StataCorp.).

## Results

### Clinicopathologic Characteristics

A total of 105 patients were included, with a median age of 62 years (range, 44‐87) and a predominance of males (102/105; 97.1%). Nearly all tumors were conventional squamous cell carcinoma (103/105; 98.1%), with 2 sarcomatoid variants. The mean tumor size was 3.5 ± 1.5 cm (median, 3.0; range, 1.0‐8.0 cm). Most patients presented with advanced local disease: 73.3% (77/105) were staged T3‐T4, with T4a the most frequent single category (46/105; 43.8%); an additional 10 specimens (9.5%) were reported as T4 without an a/b subcategory. Twenty‐eight patients (26.7%) had T1‐T2 disease. One patient was staged T3 despite histopathologic evidence of TGI, representing a misclassification under AJCC/NCCN criteria.^3^, ^11^ Nodal status was negative in 39.0% (41/105), positive in 21.9% (23/105), and undocumented in 39.0% (41/105). One patient had distant metastasis.

Bilateral involvement of at least 1 laryngeal subsite was present in 76.2% (80/105), while 23.8% had unilateral disease. Supraglottic, glottic, and subglottic involvement occurred in 74.3%, 92.4%, and 17.1% of patients, respectively. Transglottic extension was present in 66.7% (70/105), and 11.4% (12/105) had tumor involving all 3 laryngeal levels. Margins were positive in 21.0% (22/105) and thyroid cartilage invasion was present in 58.1% (61/105). Perineural invasion was present in 33.0% (31/94) and lymphovascular invasion in 25.7% (26/101) of patients with documented status. Among conventional SCC with a documented grade, 46 were well differentiated, 24 well to moderately differentiated, 25 moderately differentiated, and 1 moderately to poorly differentiated; grade was not specified in 7 reports (Table 1).

### Predictors of Thyroid Gland Invasion

TGI was identified in 8 of 105 patients (7.6%). When analysis was restricted to patients with T3‐T4 disease (n = 77), the rate of histopathologic TGI was 10.4% (8/77), compared with 7.6% in the overall cohort. No cases of TGI were observed among the 28 patients with T1‐T2 tumors (0/28). This higher invasion rate in advanced‐stage tumors underscores the anatomic relationship between extralaryngeal extension and thyroid involvement.

Tumors with TGI were significantly larger than those without (5.0 ± 1.3 vs 3.3 ± 1.4 cm; *P* = .003; probability of superiority 0.82). Thyroid cartilage invasion was present in every TGI case (100% vs 54.6%; *P* = .020; Cramér's *V* = 0.24). Subglottic involvement showed a nonsignificant trend toward increased odds (37.5% vs 15.5%; *P* = .136), as did T4 category (87.5% vs 50.5%; *P* = .065).

Bilateral laryngeal involvement (100% vs 74.2%; *P* = .194) and transglottic extension (87.5% vs 64.9%; *P* = .264) were not individually associated with TGI, and their combination likewise did not reach statistical significance (87.5% vs 47.4%; *P* = .060). Tumor involving all 3 laryngeal levels was the only pattern of laryngeal spread showing a nominal association with TGI (37.5% vs 9.3%; *P* = .047; *V* = 0.24), which persisted when combined with bilateral involvement (37.5% vs 8.2%; *P* = .036; *V* = 0.25). Given only 8 invasion events, these associations are exploratory and should be regarded as hypothesis‐generating. Histologic grade (*P* = .88), nodal status (*P* = .733), perineural invasion (*P* = .432), lymphovascular invasion (*P* = .369), and margin status (*P* = 1.000) were not significantly associated with TGI (Table 2).

**Table 2:** Clinicopathologic Predictors of Thyroid Gland Invasion

Variable	TGI (n = 8)	No TGI (n = 97)	*P* value	Cramér's *V*
Tumor size, cm (mean ± SD)	5.0 ± 1.3	3.3 ± 1.4	.003	PS = 0.82
Thyroid cartilage invasion	8/8 (100)	53/97 (54.6)	.020	0.24
Bilateral laryngeal involvement	8/8 (100)	72/97 (74.2)	.194	0.16
Subglottic involvement	3/8 (37.5)	15/97 (15.5)	.136	0.16
Transglottic extension (supraglottis + glottis)	7/8 (87.5)	63/97 (64.9)	.264	0.13
Transglottic + bilateral involvement	7/8 (87.5)	46/97 (47.4)	.060	0.21
Involvement of all 3 laryngeal levels	3/8 (37.5)	9/97 (9.3)	.047	0.24
All 3 levels + bilateral involvement	3/8 (37.5)	8/97 (8.2)	.036	0.25
T3‐T4 category	8/8 (100)	69/97 (71.1)	.106	0.17
T4 category (any)	7/8 (87.5)	49/97 (50.5)	.065	0.20
Histologic grade (documented SCC)			.88	0.08
Well differentiated	2/6 (33.3)	44/90 (48.9)		
Well to moderately differentiated	2/6 (33.3)	22/90 (24.4)		
Moderately differentiated	2/6 (33.3)	23/90 (25.6)		
Moderately to poorly differentiated	0/6 (0)	1/90 (1.1)		
Nodal status			.733	0.08
Negative	3/8 (37.5)	38/97 (39.2)		
Positive	1/8 (12.5)	22/97 (22.7)		
Undocumented	4/8 (50.0)	37/97 (38.1)		
Perineural invasion (documented)	4/8 (50.0)	27/86 (31.4)	.432	0.11
Lymphovascular invasion (documented)	3/7 (42.9)	23/94 (24.5)	.369	0.11
Positive margins	1/8 (12.5)	21/97 (21.6)	1.000	0.06
Total thyroidectomy performed	6/8 (75.0)	28/97 (28.9)	.013	0.26

Values are n/N (%) unless otherwise indicated. *P* values are from the Fisher exact test for categorical variables and the Wilcoxon‐Mann‐Whitney rank‐sum test for tumor size. Effect size is Cramér's *V*, except for tumor size, for which the probability of superiority is reported. Odds ratios are not reported because zero and near‐zero cell counts render them unstable. Transglottic extension was defined as tumor involving both the supraglottis and the glottis; involvement of all 3 laryngeal levels denotes tumor involving the supraglottis, glottis, and subglottis. Given only 8 invasion events, all comparisons are exploratory and hypothesis‐generating, and multivariable adjustment was not feasible.

Abbreviations: PS, probability of superiority; SCC, squamous cell carcinoma; SD, standard deviation; TGI, thyroid gland invasion; *V*, Cramér's *V*.

### Surgical Management and Postoperative Hypocalcemia

Partial thyroidectomy was performed in 67.6% (71/105) of patients and total thyroidectomy in 32.4% (34/105). Patients with TGI were more likely to undergo total thyroidectomy than those without invasion (75.0% [6/8] vs 28.9% [28/97]; *P* = .013; *V* = 0.26) (Figure 1). Postoperative hypocalcemia occurred in 41.9% (44/105) of patients overall and was significantly more frequent after total thyroidectomy than after partial thyroidectomy (79.4% [27/34] vs 23.9% [17/71]; *χ* ^2^ = 29.06, *P* < .001; Cramér's *V* = 0.53) (Figure 2). Intravenous calcium replacement was required in 19.0% (20/105) of patients overall, and was needed far more often after total thyroidectomy than after partial thyroidectomy (47.1% [16/34] vs 5.6% [4/71]; *χ* ^2^ = 25.59, *P* < .001; Cramér's *V* = 0.49). Among the 44 patients who developed hypocalcemia, intravenous replacement was required in 59.3% (16/27) of those who had undergone total thyroidectomy versus 23.5% (4/17) of those who had undergone partial thyroidectomy (*χ* ^2^ = 5.37, *P* = .021; *V* = 0.35) (Figure 3).

**Figure 1:**
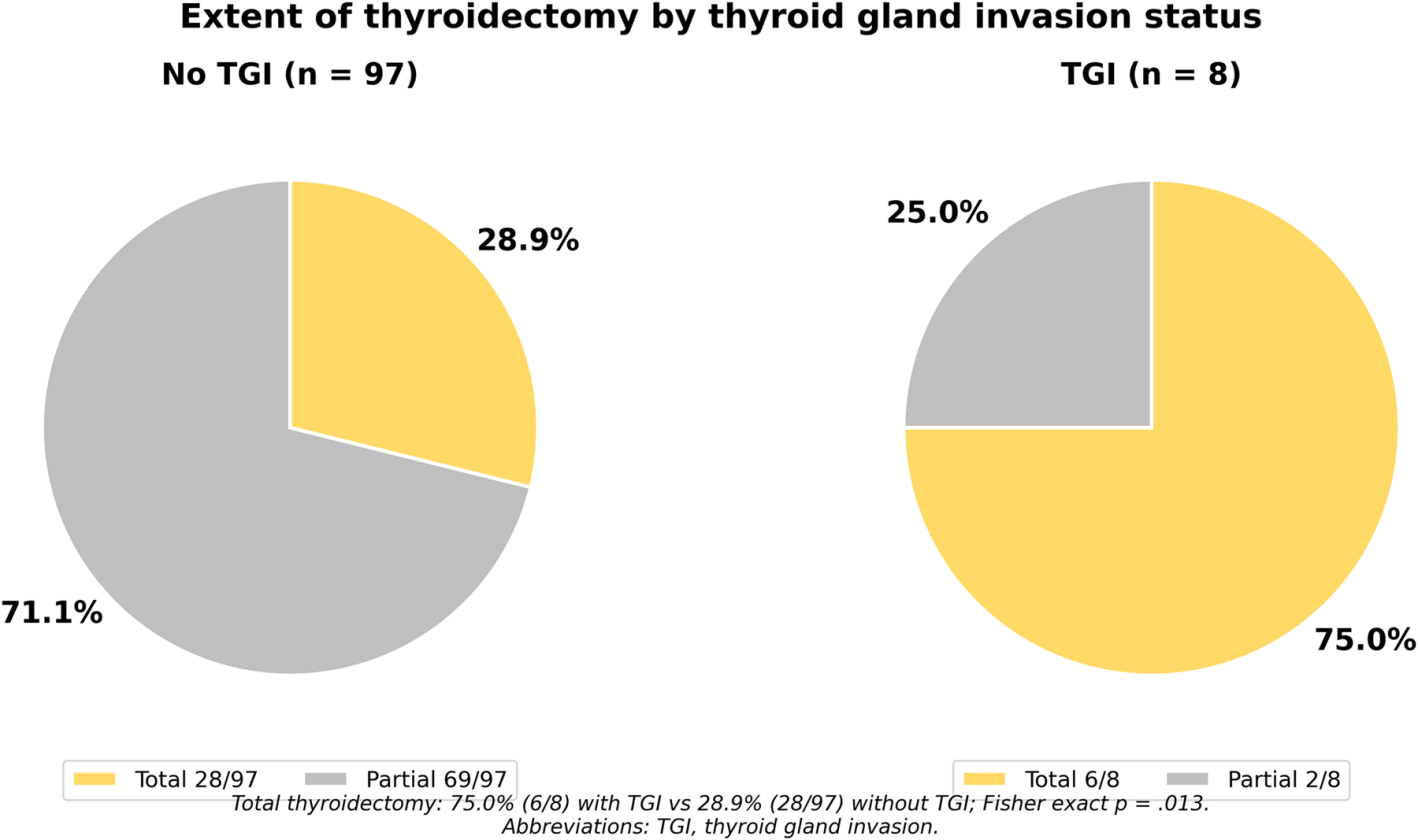
Extent of thyroidectomy by thyroid gland invasion (TGI) status. Patients with TGI (n = 8) were significantly more likely to undergo total thyroidectomy than patients without TGI (n = 97) (75.0% [6/8] vs 28.9% [28/97]; Fisher exact *P* = .013; Cramér's *V* = 0.26).

**Figure 2:**
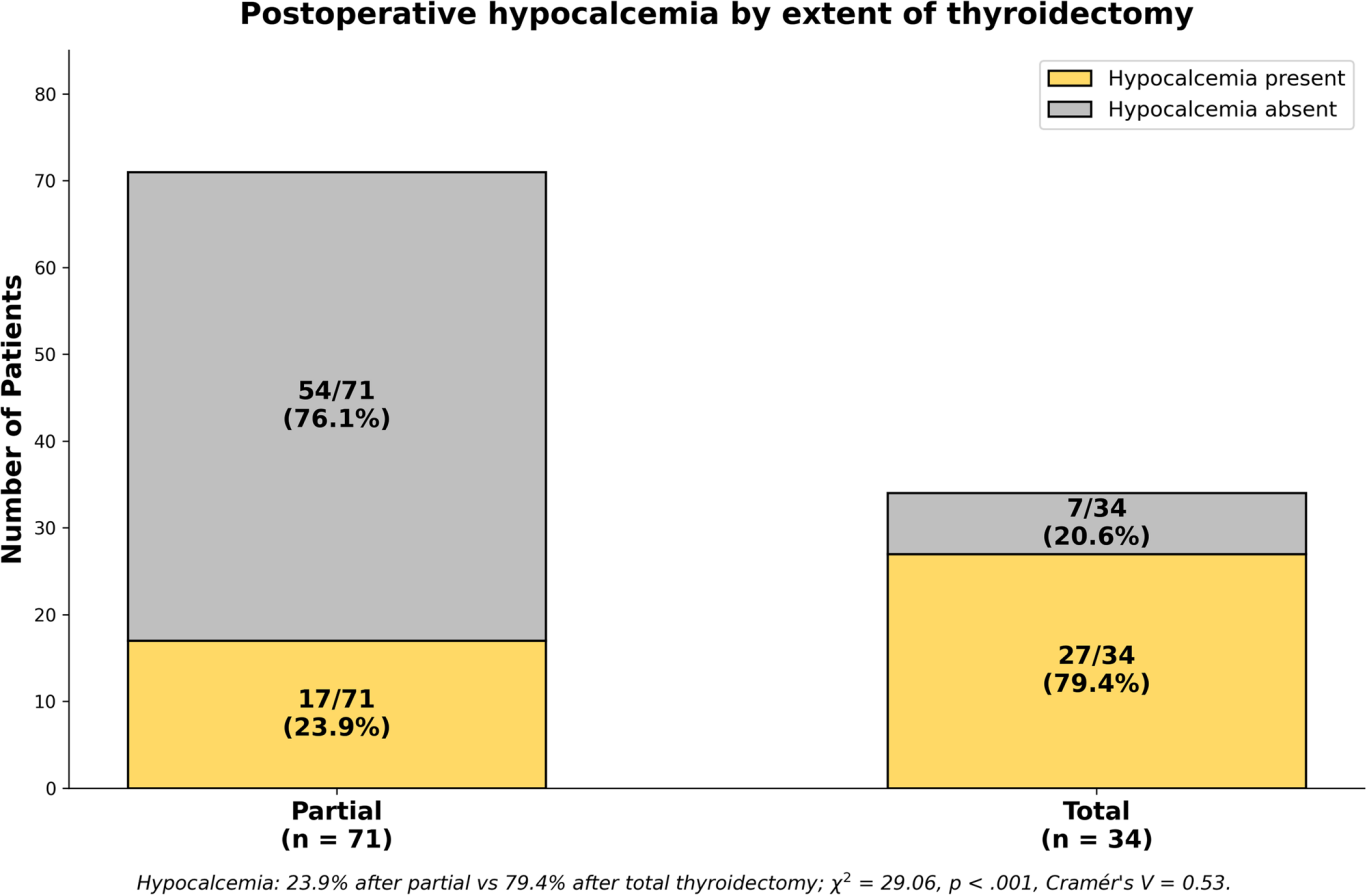
Postoperative hypocalcemia stratified by extent of thyroidectomy. Hypocalcemia occurred in 41.9% (44/105) of the surgical cohort, with significantly higher frequency after total thyroidectomy than after partial thyroidectomy (79.4% [27/34] vs 23.9% [17/71]; *χ* ^2^ = 29.06, *P* < .001; Cramér's *V* = 0.53).

**Figure 3:**
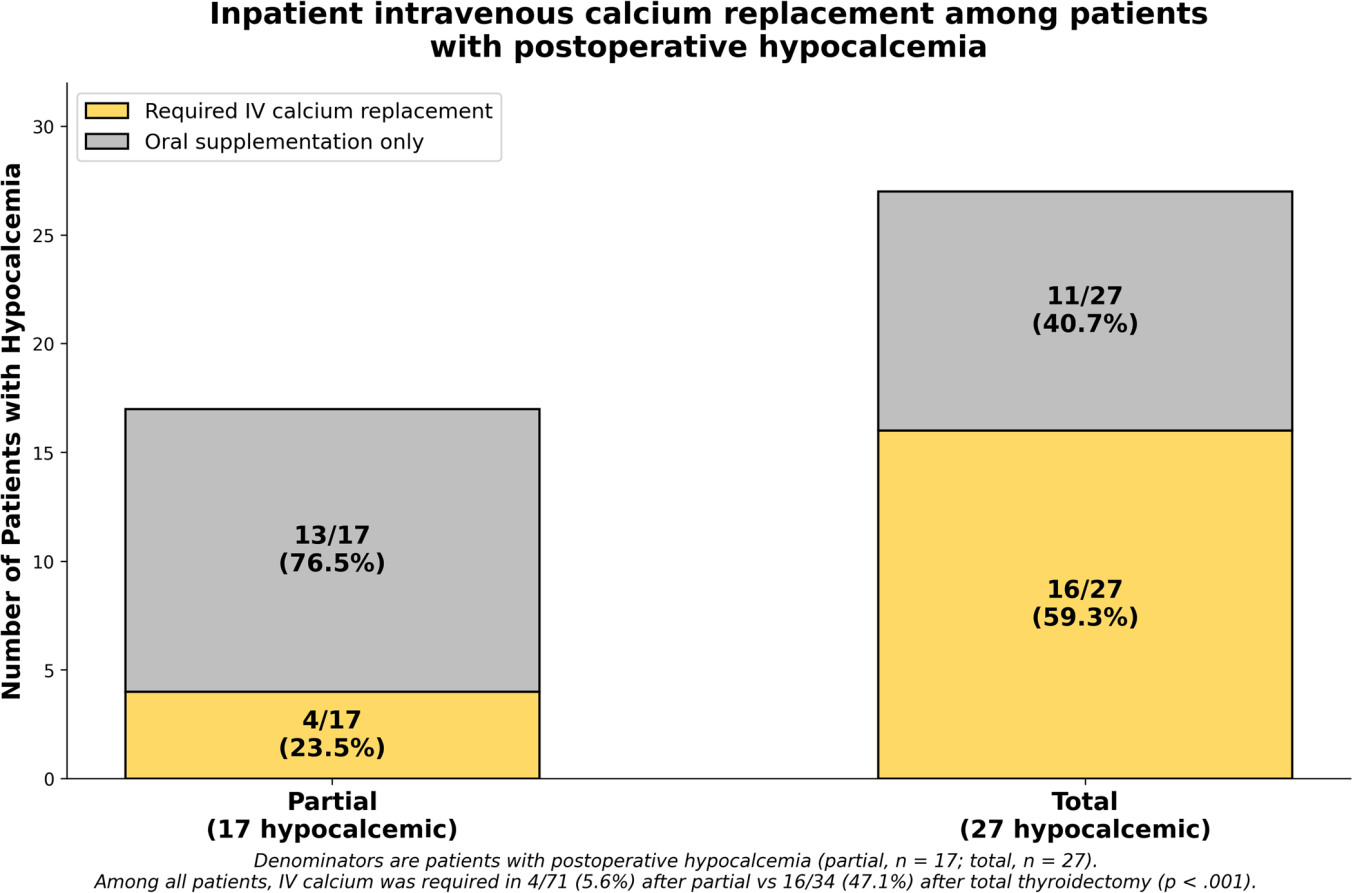
Inpatient intravenous (IV) calcium replacement among the 44 patients who developed postoperative hypocalcemia. Denominators are patients with hypocalcemia in each surgical group. Escalation to IV replacement was more frequent after total thyroidectomy than after partial thyroidectomy (59.3% [16/27] vs 23.5% [4/17]; *χ* ^2^ = 5.37, *P* = .021; Cramér's *V* = 0.35). Considering the whole cohort, IV calcium was required in 47.1% (16/34) of total thyroidectomy patients versus 5.6% (4/71) of partial thyroidectomy patients (*P* < .001).

## Discussion

In this single‐institution cohort of 105 patients undergoing total laryngectomy, histologic thyroid gland invasion was identified in 8 patients (7.6%). On univariate analysis, TGI was significantly associated with larger tumor size and thyroid cartilage involvement. These findings confirm that while TGI is uncommon overall, it occurs predominantly in anatomically advanced tumors. Given the limited number of invasion events, multivariate regression was not feasible; nonetheless, the observed associations are consistent with those reported in prior studies.

An important refinement in this revision concerns the patterns of laryngeal spread. Bilateral involvement and transglottic extension were not individually associated with TGI (*P* = .194 and *P* = .264, respectively), and—contrary to what a preliminary analysis suggested—their combination also failed to reach statistical significance (*P* = .060). Only tumor extending across all 3 laryngeal levels showed a nominal association with TGI (*P* = .047), an association that persisted when combined with bilateral disease (*P* = .036). We therefore do not claim that bilateral involvement or transglottic extension, individually or in combination, predicts TGI in this cohort. With only 8 invasion events, these null results should be interpreted cautiously and may represent type II error rather than true absence of association; equally, the borderline association observed for 3‐level involvement rests on 3 events and requires independent confirmation. Larger multicenter studies are necessary to definitively evaluate these potential predictors.

The inclusion of 28 patients with T1‐T2 disease in our cohort—none of whom demonstrated TGI—warrants discussion. This likely reflects salvage laryngectomy cases (radiation failures) and other clinical scenarios necessitating total laryngectomy despite early T‐stage classification. Importantly, by AJCC definition, the presence of TGI automatically upstages a tumor to T4a, rendering invasion anatomically implausible in true early‐stage disease where cartilage integrity remains intact. When analysis was restricted to T3‐T4 patients, the TGI rate rose to 10.4% (8/77), more accurately reflecting risk in patients with advanced local disease. Notably, even at this higher rate, nearly 90% of high‐risk patients did not harbor thyroid involvement, supporting selective rather than routine thyroidectomy.

Our TGI incidence of 7.6% aligns closely with prior reports ranging from 6% to 12%, including the largest monocentric series by Gorphe et al^12^ (9.9%, n = 484) and the pooled systematic review estimate of 8.08% by Xie et al.^13^ Predictors identified in these series—including subglottic involvement, T4 stage, full‐thickness cartilage invasion, and anterior soft‐tissue extension^4^, ^12^, ^13^, ^14^—were partially corroborated in our cohort, with significant associations for tumor size and cartilage involvement and a nonsignificant trend for subglottic involvement and T4 category. Our findings are also consistent with anatomic studies describing preferential pathways of anterior and inferior tumor spread toward the thyroid gland via cricothyroid membrane penetration and anatomic defects in the thyroid cartilage framework.^8^, ^9^

Historically, routine ipsilateral thyroid lobectomy was performed during laryngectomy regardless of thyroid involvement.^15^ Contemporary evidence, however, supports a selective, risk‐adapted approach. Thyroidectomy in the absence of invasion confers no oncologic benefit while exposing patients to substantial endocrine morbidity. Our findings align with current recommendations to reserve thyroidectomy for patients with high‐risk features—including T4 stage, large tumor size, extensive cartilage involvement, or radiologic/intraoperative evidence of extralaryngeal extension—while prioritizing thyroid preservation in lower‐risk cases.^11^, ^12^, ^13^, ^14^, ^15^, ^16^

The magnitude of postoperative endocrine morbidity in our cohort was substantial. Total thyroidectomy was associated with a more than threefold increase in hypocalcemia compared with partial resection (79.4% vs 23.9%; *P* < .001), and intravenous calcium replacement was required in 47.1% of total thyroidectomy patients versus 5.6% after partial resection (*P* < .001). Among patients who did become hypocalcemic, those who had undergone total thyroidectomy were also more likely to require escalation to intravenous therapy (59.3% vs 23.5%; *P* = .021), indicating that total thyroidectomy increases not only the frequency but also the severity of hypocalcemia. These findings corroborate prior reports by Gal et al,^5^ Negm et al,^6^ and Basheeth et al,^7^ which collectively underscore the substantial risks of parathyroid dysfunction and hypocalcemia following thyroid resection during laryngectomy. However, the relatively high rate of hypocalcemia observed even after partial thyroidectomy (23.9%) is notable and may reflect several factors, including concomitant central compartment neck dissection (which was not assessed in our retrospective review), inadvertent transient parathyroid devascularization or injury, and possible inclusion of post‐radiation salvage cases with compromised parathyroid blood supply. The lack of detailed intraoperative documentation regarding neck dissection extent and parathyroid gland management represents a limitation of our study.

These endocrine complications carry significant clinical consequences extending beyond laboratory abnormalities. Hypocalcemia can manifest as paresthesias, tetany, seizures, and life‐threatening cardiac arrhythmias, while hypothyroidism may contribute to impaired wound healing, cardiovascular dysfunction, pharyngocutaneous fistula formation, and neuropsychiatric sequelae including depression.^17^ In a patient population often burdened by multiple medical comorbidities and tobacco‐related disease, the cumulative impact of endocrine dysfunction is considerable and necessitates lifelong monitoring, replacement therapy, and specialized follow‐up.^12^, ^13^, ^14^, ^15^, ^16^

### Limitations

This study has several important limitations. First, the retrospective, single‐institution design is inherently subject to selection bias and incomplete data capture. Preoperative imaging findings, which could inform risk stratification, were inconsistently available. Nodal status was undocumented in 39.0% of cases, potentially limiting our ability to detect associations with TGI, and perineural and lymphovascular invasion were not reported in 11 and 4 pathology reports, respectively. Second, the small number of invasion events (n = 8) substantially reduced statistical power and precluded multivariate analysis, thereby limiting our ability to adjust for confounders and identify independent predictors. Third, intraoperative findings—including gross extralaryngeal extension, cricothyroid membrane involvement, and anterior framework invasion—were not systematically recorded despite their reported predictive value in other series.^12^, ^14^

Fourth, the positive margin rate of 21.0% (22/105) is higher than optimal and warrants explanation. This likely reflects the advanced stage at presentation (73.3% with T3‐T4 disease), potential inclusion of salvage laryngectomy cases with radiation‐induced tissue fibrosis, and socioeconomic barriers to early diagnosis in our medically underserved population. Detailed intraoperative margin assessment protocols, including frozen section utilization, were not consistently documented and could not be systematically analyzed.

Fifth, we were unable to differentiate between primary and salvage (postradiation) laryngectomy cases due to incomplete radiation therapy documentation following electronic health record migration. This distinction is clinically important because prior radiation fundamentally alters tissue characteristics—increasing fibrosis, obscuring anatomic planes, and compromising parathyroid vascularity—all of which may influence both thyroid invasion patterns and postoperative hypocalcemia rates. Consequently, the hypocalcemia rates observed in our cohort may reflect a heterogeneous mixture of primary and salvage cases, and we cannot determine whether the elevated rates after total thyroidectomy are partially attributable to prior radiation exposure. Future prospective studies with complete treatment histories are needed to stratify these populations and assess their differential risks.

Sixth, our pathology reports lacked consistent documentation of the laterality of thyroid invasion. Laterality was specified in only 2 of the 8 TGI cases. While TGI occurred exclusively in patients with bilateral laryngeal disease, we could not determine in most cases whether invasion itself was unilateral (ipsilateral to the dominant tumor) or bilateral. This distinction has important clinical implications: if bilateral laryngeal tumors predominantly result in unilateral thyroid invasion, hemithyroidectomy may suffice even with bilateral laryngeal involvement, thereby avoiding unnecessary contralateral lobe resection and reducing hypothyroidism and hypocalcemia risk. The observation that 75.0% of TGI patients underwent total thyroidectomy suggests surgical decision‐making may have been influenced by thyroid cartilage involvement and gross intraoperative findings rather than confirmed bilateral thyroid invasion. Future studies should incorporate systematic intraoperative assessment and pathologic documentation of invasion laterality to optimize surgical decision‐making.

Seventh, long‐term endocrine outcomes—including resolution of hypocalcemia, duration of calcium and vitamin D supplementation, and rates of permanent hypoparathyroidism—were not systematically documented and could not be evaluated. These outcomes are critical for comprehensive patient counseling regarding the full burden of thyroidectomy and represent an important priority for future prospective investigation.

Eighth, detailed data on postoperative parathyroid hormone (PTH) levels, supplementation regimens (calcium and vitamin D dosing), and clinical/laboratory criteria for escalating to intravenous calcium therapy were not systematically documented. Parathyroid gland tissue was inconsistently documented in final pathology reports, likely reflecting underreporting rather than true absence, as small parathyroid glands may not be routinely identified unless specifically sought during gross examination. This reflects both the retrospective study design and the evolution of clinical practice across the 13‐year study period. In addition, our study focused on the prevalence of TGI and immediate postoperative hypocalcemia rates. Detailed assessment of intraoperative parathyroid gland management strategies (including autotransplantation) and long‐term calcium homeostasis outcomes were beyond the scope of this investigation. Future prospective studies specifically designed to evaluate parathyroid preservation techniques and their impact on permanent hypoparathyroidism rates would provide valuable complementary data.

Finally, oncologic outcomes including disease‐free survival, overall survival, and recurrence patterns were not retrievable from our retrospective review. This prevents evaluation of the long‐term prognostic significance of TGI and precludes assessment of whether thyroid preservation impacts locoregional control or survival. Understanding these relationships would substantially inform surgical decision‐making and represents an essential direction for future prospective research with systematic long‐term follow‐up.

Despite these limitations, several factors support the validity and value of our findings. First, our results demonstrate consistency with established anatomic pathways and clinical predictors identified in larger series, suggesting our cohort reflects broader biological patterns. Second, this study represents one of the largest single‐institution analyses of thyroid invasion in a Hispanic population—a significantly underrepresented group in head and neck oncology literature. Our demonstration that TGI epidemiology and risk factors in Puerto Rican Hispanic patients align with predominantly non‐Hispanic cohorts supports the generalizability of selective thyroid management strategies across diverse populations and addresses an important gap in health disparities research.

## Conclusion

TGI in LSCC was uncommon in this single‐institution Hispanic cohort, occurring in 7.6% of patients overall and 10.4% of those with T3‐T4 disease. TGI was associated with larger tumors (mean, 5.0 cm) and with thyroid cartilage involvement, and showed non‐significant trends with subglottic involvement and T4 category. Among patterns of laryngeal spread, only extension across all 3 laryngeal levels showed a nominal association with TGI; bilateral involvement and transglottic extension were not associated with TGI, either individually or in combination. These findings reinforce that thyroid gland invasion is concentrated in anatomically advanced disease rather than being an inherent feature of laryngeal cancer. Oncologic outcomes such as recurrence and survival were not retrievable, preventing evaluation of the long‐term prognostic significance of TGI or of the extent of thyroidectomy. Future prospective studies with systematic long‐term oncologic follow‐up are needed to address this question.

Importantly, the extent of thyroid surgery had a major impact on postoperative morbidity: total thyroidectomy was associated with substantially higher rates and greater severity of hypocalcemia, with intravenous calcium replacement required roughly eight times as often as after partial thyroidectomy (47.1% vs 5.6%), underscoring the increased burden of endocrine dysfunction when the entire gland is removed.

Given the impact of hypocalcemia on quality of life and the risk of permanent endocrine dysfunction, these results highlight the clinical importance of tailoring thyroid management during total laryngectomy.

Our data therefore support a selective approach: reserving thyroid resection for clearly high‐risk presentations (T4 disease, large tumor size, thyroid cartilage invasion, or convincing preoperative radiologic and/or intraoperative evidence of extralaryngeal involvement), while sparing the thyroid in the absence of such features. Adoption of evidence‐based selection criteria has the potential to reduce endocrine complications without compromising oncologic safety and to improve patients' quality of life.

Finally, the inclusion of a Hispanic cohort contributes valuable evidence from an underrepresented minority population, broadening the generalizability of these findings and addressing gaps in the literature. Larger multicenter studies with long‐term oncologic outcomes are needed to validate predictive factors, refine patient selection, and establish standardized guidelines for thyroid management in LSCC treated with total laryngectomy.

## Author Contributions

**Francisco J. Garratón‐Gutiérrez**, conduct, writing, revision, presentation; **Bianca Bonafont‐Reyes**, conduct, writing, revision; **Javier A. Vilá Ortiz**, conduct, writing, revision; **Antonio Burés‐Rodríguez**, conduct, revision; **Mario Corona‐Ruíz**, design; **Jeamarie Pascual‐Marrero**, design, revision.

## Disclosures

### Competing interests

None.

### Funding source

This study was supported by the UPR School of Medicine Endowed Health Services Research Center, Awards Number 5S21MD000242 and 5S21MD000138 from the National Center for Minority Health and Health Disparities of the National Institutes of Health (NCMHD‐NIH). The content is solely the responsibility of the authors and does not necessarily represent the official views of the NCMHD or NIH.
